# Assessing the Readability of Online Patient Education Materials in Obstetrics and Gynecology Using Traditional Measures: Comparative Analysis and Limitations

**DOI:** 10.2196/46346

**Published:** 2023-08-30

**Authors:** Anunita Nattam, Tripura Vithala, Tzu-Chun Wu, Shwetha Bindhu, Gregory Bond, Hexuan Liu, Amy Thompson, Danny T Y Wu

**Affiliations:** 1 College of Medicine University of Cincinnati Cincinnati, OH United States; 2 Department of Computer Science University of Cincinnati Cincinnati, OH United States; 3 School of Criminal Justice University of Cincinnati Cincinnati, OH United States

**Keywords:** obstetrics and gynecology, online patient education materials, readability, online education, education, health literacy, literature, medical documents, tool, utilization, online content, health education, education material, assessment, obstetrics, gynecology

## Abstract

**Background:**

Patient education materials (PEMs) can be vital sources of information for the general population. However, despite American Medical Association (AMA) and National Institutes of Health (NIH) recommendations to make PEMs easier to read for patients with low health literacy, they often do not adhere to these recommendations. The readability of online PEMs in the obstetrics and gynecology (OB/GYN) field, in particular, has not been thoroughly investigated.

**Objective:**

The study sampled online OB/GYN PEMs and aimed to examine (1) agreeability across traditional readability measures (TRMs), (2) adherence of online PEMs to AMA and NIH recommendations, and (3) whether the readability level of online PEMs varied by web-based source and medical topic. This study is not a scoping review, rather, it focused on scoring the readability of OB/GYN PEMs using the traditional measures to add empirical evidence to the literature.

**Methods:**

A total of 1576 online OB/GYN PEMs were collected via 3 major search engines. In total 93 were excluded due to shorter content (less than 100 words), yielding 1483 PEMs for analysis. Each PEM was scored by 4 TRMs, including Flesch-Kincaid grade level, Gunning fog index, Simple Measure of Gobbledygook, and the Dale-Chall. The PEMs were categorized based on publication source and medical topic by 2 research team members. The readability scores of the categories were compared statistically.

**Results:**

Results indicated that the 4 TRMs did not agree with each other, leading to the use of an averaged readability (composite) score for comparison. The composite scores across all online PEMs were not normally distributed and had a median at the 11th grade. Governmental PEMs were the easiest to read amongst source categorizations and PEMs about menstruation were the most difficult to read. However, the differences in the readability scores among the sources and the topics were small.

**Conclusions:**

This study found that online OB/GYN PEMs did not meet the AMA and NIH readability recommendations and would be difficult to read and comprehend for patients with low health literacy. Both findings connected well to the literature. This study highlights the need to improve the readability of OB/GYN PEMs to help patients make informed decisions. Research has been done to create more sophisticated readability measures for medical and health documents. Once validated, these tools need to be used by web-based content creators of health education materials.

## Introduction

Readability assessment of online health information (OHI) provides valuable insight into how much information is easily understandable, to effectively empower laypersons to make informed health decisions [1]. Specifically, recognizing the impact of the situational reading level demands of the population and implementing that knowledge to simplify OHI, particularly materials geared toward patient education, can act as a mediating factor in health and clinical decision-making for patients by simplifying clinician-patient communication and reducing the complexities of the health care system for the patient [2]. The aim of online patient education materials (PEMs) should be to support interactive health literacy within an individual, giving them the ability to extract health information and derive meaning from different sources. This ability grants patients the opportunity to engage in interactions with health care professionals, fostering greater understanding and shared decision making. Therefore, using the internet population’s reading level and literacy capacity as a metric to guide PEMs can increase the usability and effectiveness of PEMs [3]. Furthermore, as the internet now serves as the primary source of information in modern society, and the prevalence of smartphones has allowed the expansion of the internet to a wider population, improving the quality of OHI and PEMs can reach a diversity of populations, and can decrease the burdens of frontline professional support and patients alike [4].

Current recommendations made by the American Medical Association (AMA) and the National Institutes of Health (NIH) suggest that all PEMs should be at a sixth-eighth–grade reading level or lower [5,6]. However, previous studies conducted broadly throughout clinical specialties indicate these guidelines are not regularly followed [7]. Moreover, our preliminary scoping review conducted prior to this study showed that there is a lack of readability assessment studies in the field of obstetrics and gynecology (OB/GYN). Since OB/GYN PEMs found on the internet are often accessed to seek guidance on a wide range of symptoms, diagnoses, and treatments, it provides patients with information on a spectrum of preventable and curable gynecological diseases. Of note, 1 important public health example is human papillomavirus and cervical cancer, for which preventative care via vaccination and early detection through Papanicolaou test screening are available. Given that OB/GYN is a primary health care field, lack of access to appropriate online PEMs may be a limiting factor in overcoming poor interactive health literacy and its associated outcomes [8].

Over recent years, popular traditional readability measures (TRMs), such as the Flesch-Kincaid grade level (FKGL) [9], Gunning fog index (GFI) [10], Simple Measure of Gobbledygook (SMOG) [11], and the Dale-Chall (DCL) [12] formulas generate a grade-level score associating readability with the grade level of education needed to understand a document. For example, a document with a readability of the 5th-grade level is easier to read and understand than a document with a 10th-grade reading level. The TRMs used similar textual features in their formula (Table 1), including the average number of words per sentence, the average number of syllables per word, the average number of sentences, and custom easy versus difficult word lists.

Previous research done to assess the readability of OB/GYN PEMs, although scarce, has shown that most existing OB/GYN PEMs are written between the 9th and 12th grade level [13-18]. However, several of these studies focus solely on PEMs published by academic sources, limiting the study to a specific subtype of PEMs that patients are likely to encounter on the internet [13,14]. Moreover, in these studies where TRMs were used, scores generated by each measure were not always consistent, indicating a limitation related to the validity of these TRMs used to widely assess patient readability and understanding [19].

Therefore, to address the gaps in existing research and further address the issue of readability in online OB/GYN PEMs, 4 research questions were produced. First, do the scores generated by 4 TRMs agree with each other (research question 1 [RQ1])? This research question was prompted by previous research indicating that certain readability measures do not agree with each other, particularly FKGL, SMOG, and GFI [20-22]. Second, do the PEMs found in the field of OB/GYN follow the sixth-eighth–grade level readability recommendation by the AMA and NIH (research question 2 [RQ2])? Third, are there differences in readability level by sources (government agencies, nonprofit organizations, educational websites, or commercial entities) (research question 3 [RQ3])? Finally, will the readability level change when discussing different gynecological processes and topics, such as menstruation, pregnancy, cancer, general disease, and procedural information (research question 4 [RQ4])?

**Table 1:** Mathematical equation used for each TRM.^a^

Formula	Equation
Flesch-Kincaid grade level (FKGL) [9]	Reading grade level = (0.39 × average number of words per sentence) + (11.8 × average number of syllables per word) − 15.59
Gunning fog index (GFI) [10]^b^	Reading grade level = 0.4 (average number of words per sentence + number of words with 3 or more syllables × [100 / number of words])
Simple Measure of Gobbledygook (SMOG) [11]	If number of sentences ≥ 30: reading grade level = 3 + square root of polysyllable count to the nearest perfect square. Else if number of sentences is between 1 and 29: raw score = average number of polysyllable per sentence × ratio of sentences) + number of polysyllable Round the Raw score to the integer. Reading grade level = 5 if Raw score is between 1 and 6 = R if Raw score is in [R^2^ − 7 × R + 13, (R − 2) × (R − 3)] where R is between 6 and 17 = 18 if Raw score is equal to or larger than 211
Dale-Chall (DCL) [12]	Raw score = (0.0496 × average number of words per sentence) + (15.79 × number of words not found on a word list) / (number of words) + 3.6365 Reading grade level = 4 if Raw score is smaller than 5 = 2 × Raw score − 5 if Raw score is between 5 and 8 = 3 × Raw score − 14 if Raw score is 9 = 16 if Raw score is equal to or larger than 10

^a^TRM: traditional readability measures.

^b^Requires at least 100 words in the input document.

## Methods

### Data Collection and Coding

**Figure 1:**
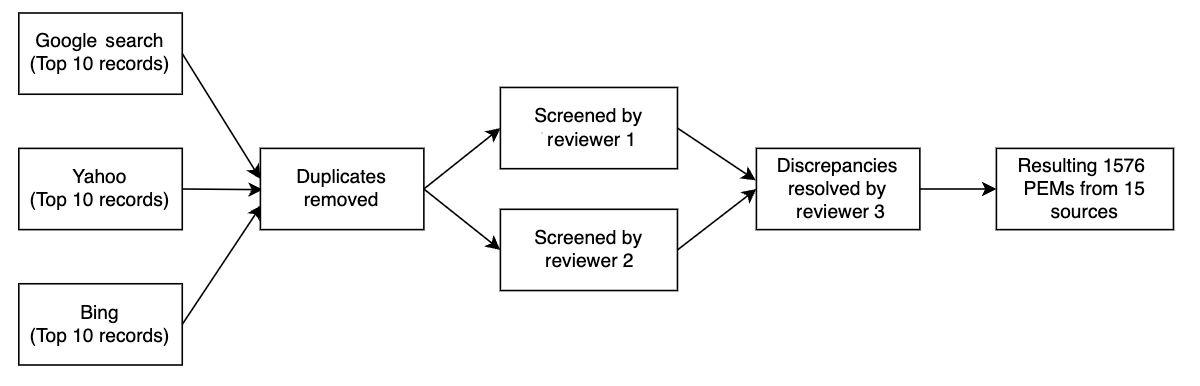
Data collection and screening process. PEM: patient education material.

The PEMs were selected by searching the keywords “OB/GYN” AND “Patient Education Materials” in the 3 most used search engines (ie, Google, Yahoo, and Bing!), and selecting the results on the first page (top 10 results) from each. This is not considered as a scoping review. Patient search behavior and patterns were mimicked in order to generate the most realistic search results. After removing repetitions and broken links, 15 different website sources from varying categories were identified containing 1576 PEMs (Multimedia Appendix 1). The PEMs were crawled by using the Python Selenium library, which automatically visited the individual web pages and extracted the full text for each paper with formatting retained. In terms of coding, 2 reviewers (TV and Somya Pandey) surveyed the texts together and coded a small sample to determine the codebook by sources and topics. This process is considered as a bottom-up approach as opposed to a top-down approach using predefined categories. Then, the 2 reviewers coded all the PEMs independently to produce the source and topic categories. Any discrepancy was resolved by the third reviewer (AN). Of note, each PEM might have multiple topics. The primary topic of each PEMs was determined and used for the subsequent statistical analysis. The process of data collection and screening is shown in Figure 1.

### Readability Scoring

Among the 1576 PEMs crawled, 93 of them did not have enough number of words for GFI scoring (minimum 100 words). These PEMs were removed in the readability scoring and subsequent analysis, resulting in a total of 1483 OB/GYN PEMs analyzed. To obtain readability scores, the text of the collected PEMs was first cleaned, primarily by removing the title and any sections unrelated to the health material, such as mentions of other papers and advertisements, and then scored based on the TRM formulas. Specifically, each PEM was broken down into sentences, words, and syllables. Sentences were derived by splitting the text by common end-of-sentence punctuation, while words were found by using a space delimiter for each sentence; syllables and base words were found mainly through the Natural Language Tool Kit Python library. The Natural Language Tool Kit Python library is a collection of modules to process text and perform natural language processing (NLP) tasks [23]. Surface metrics of the text, such as the average number of words per sentence, were then calculated based on the 4 TRMs to generate the scores. Of note, the DCL raw scores were transformed into grade levels using its reading grade level formula (Table 1). Only the integer part of the result from the mathematical equations will be preserved as the reading grade level. A random sample of 50 PEMs was selected and hand calculations were conducted and compared to the Python-generated values to ensure validity of our methods. Of note, the decision to use these 4 TRMs (FKGL, GFI, SMOG, and DC) was based on their use in previous studies assessing the readability of online PEMs, although not particularly in the field of OB/GYN [24-27].

### Data Analysis

To address our research questions, respective hypotheses were proposed. First (RQ1), it was hypothesized that the TRMs would have acceptable agreeability (hypothesis 1) since they used similar textual features and determined readability as grade levels. To test this hypothesis, each readability score of a document was converted to a grade level from grade 4 to 16 due to the grade level mapping scale of DCL and treated as categorical data. In addition, the study tried to improve the agreement by combining the categories, for example, less than sixth grade, sixth-eighth–grade, and above eighth grade. The Fleiss κ was calculated to measure the degree of agreement between the different readability measures over that which would be expected by chance [28]. The given κ value can be a negative number up to 1.00, with negative numbers indicating poor agreement, 0.00-0.20 as slight agreement, 0.21-0.40 as fair agreement, 0.41-0.60 as moderate agreement, 0.61-0.80 as substantial agreement, and 0.81-1.00 being almost perfect to perfect agreement. When hypothesis 1 is held, meaning all 4 TRMs agree with each other substantially, FKGL will be used to represent the readability of a document since it is widely used (eg, FKGL is implemented in Word [Microsoft Corp]). Otherwise, a composite score (average or median of all 4 TRMs) will be created to represent the readability of a document.

Second (RQ2), we hypothesized that the PEMs would not follow the recommended guidelines of sixth-eighth–grade level readability (hypothesis 2) since literature of readability assessment has shown this trend. To test this hypothesis, the composite scores were used to generate both categorical and numerical data. The normality of the composite scores was examined using the Shapiro-Wilk test. The null hypothesis of this test is to assume that the population of data is normally distributed. Since the significant value of the Shapiro-Wilk test on our data was less than 0.05, the population of data deviates significantly from a normal distribution. Therefore, Wilcoxon Sign test was used to examine if the composite scores overall are above the recommended level (eighth grade).

**Figure 2:**
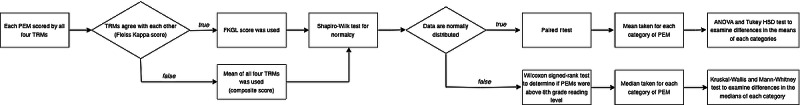
Process of data analysis. FKGL: Flesch-Kincaid grade level; HSD: honestly significant difference; PEM: patient education material; TRM: traditional readability measure.

Lastly, the study explored the possibility of differences in readability scores for PEMs coming from varying sources (RQ3) and topics (RQ4). It was hypothesized that the government sourced PEMs would have the lowest readability level (hypothesis 3), making them the easiest to understand, since the government agencies claimed that their online PEMs have been curated and are suitable for the public. On the other hand, it was hypothesized that the readability level of PEMs with different topics was similar (hypothesis 4). To test these 2 hypotheses, the PEMs were manually categorized into 4 source categories (ie, government, commercial, nonprofit, and educational) and 5 topic categories (general disease, pregnancy, menstruation, procedure, and cancer). General disease referred to any papers referencing diseases that do not affect women exclusively, cannot be placed into any of the other categories, and appeared on these OB/GYN PEM websites. The Kruskal-Wallis test was applied to detect any differences between medians in each category (main effect), followed by the pairwise Mann-Whitney test with Bonferroni correction as the post hoc test to assess whether the median of the composite scores of the different categories were statistically different (Figure 2). The median of the composite score was used because the composite scores were not normally distributed. Table 2 shows distribution of the PEMs analyzed in this study by the source and topic categorization.

**Table 2:** Contingency table of online OB/GYN PEMs^a^ sources based on topics.

Topic or source	Educational, n	Nonprofit, n	Commercial, n	Government, n	Total, n
Pregnancy	553	531	34	27	1145
General disease	39	13	57	28	137
Procedure	42	22	24	10	98
Menstruation	24	3	22	6	55
Cancer	4	1	31	12	48
Total	662	570	168	83	1483

^a^OB/GYN PEM: obstetrics and gynecology patient education material.

### Ethical Considerations

Of note, the present study did not require IRB review since it was based on publicly available records with no identifiers of individuals.

## Results

### Hypothesis 1

Placing the PEMs into 13 categories from 4 to 16, that is 1 per grade level, resulted in a very low Fleiss κ=0.0025 value, which indicates slight agreement. Placing the readability scores into 3 categories (less than sixth grade, sixth to eighth grade, and greater than eighth grade) only resulted in a slight increase in the Fleiss κ (*P*=.033) and placing them into just 2 categories (less than or equal to 8 and greater than 8) resulted in a significant increase (*P*=.08). However, the agreement is still considered slight. As a result, the 4 TRMs did not perform fair agreement in our data set. Then, the composite score of a document was generated by averaging the 4 TRMs. Figure 1 shows the distribution of the composite scores using both the mean and median. As shown in Figure 1, taking the average of the readability scores can be more conservative since the average considered a variety of responses in each TRM. On the other hand, the median involved only 2 out of 4 responses in our case, which resulted in the loss of half the information.

### Hypothesis 2

Using the composite score of the TRMs, the PEMs were found to be at the 11th grade reading level, which is significantly higher than the recommended 8th grade level (*P*<.001) using the Wilcoxon sign test. The median only was used in these calculations, as the mean was shown to be skewed by extreme values (Table 3).

**Table 3:** Distribution of the composite scores (average of the 4 TRMs^a^; RQ2^b^).

Composite readability grade level	PEMs^c^, n (%)	Accumulated number, n (%)
6	1 (0.06)	1 (0.06)
7	20 (1.27)	21 (1.33)
8	44 (2.79)	65 (4.12)
9	100 (6.35)	165 (10.47)
10	178 (11.29)	343 (21.76)
11	206 (13.07)	549 (34.84)
12	321 (20.37)	870 (55.20)
13	276 (17.51)	1146 (72.72)
14	197 (12.50)	1343 (85.22)
15	183 (11.61)	1526 (96.83)
16	50 (3.17)	1576 (100)

^a^TRM: traditional readability measures.

^b^RQ2: research question 2.

^c^PEM: patient education material.

### Hypothesis 3

The Kruskal-Wallis test showed that at least 1 median is significantly different from others (*P*<.001) among the 4 documentation sources. The pairwise Mann-Whitney test indicated that the median readability level of the government source (11.25) was significantly lower than the commercial (13.00), educational (12.75), and nonprofit (12.75). Meanwhile, the median readability level of the commercial source (13.00) was significantly higher than that of the nonprofit source (12.75), but not different from the educational source (12.75). None of the categories had a median readability level less than the recommended eighth-grade level (Table 4).

**Table 4:** Results from pairwise Mann-Whitney tests^a^ (RQ3^b^).

Source category comparison		Median of mean			
Category 1	Category 2	Median 1	Median 2	Mann-Whitney statistic	*P* value
Commercial	Government	13.00	11.25	11,223	<.01^a^
Commercial	Educational	13.00	12.75	62,131	.02
Commercial	Nonprofit	13.00	12.75	54,775	.004^a^
Government	Educational	11.25	12.75	16,356	<.01
Government	Nonprofit	11.25	12.75	15,189	<.01^a^
Educational	Nonprofit	12.75	12.75	192,935	.46

^a^*P*<.008.

^b^RQ3: research question 3.

### Hypothesis 4

The Kruskal-Wallis test showed there is at least 1 median which is significantly different from others (*P*=.001) among the 5 medical topics. The pairwise Mann-Whitney test indicated that the median readability levels of many groups were similar to each other. However, the median readability level of menstruation (13.5) and pregnancy (12.75) are significantly higher than general disease (12.25; Table 5).

**Table 5:** Results from pairwise Mann-Whitney tests^a^ (RQ4^b^).

Topic category comparison			Composite scores			
Category 1	Category 2	Median 1		Median 2	Mann-Whitney statistic	*P* value
Menstruation	Procedure	13.5		12.75	3369	.01
Menstruation	General disease	13.5		12.25	5271.5	<.01^a^
Menstruation	Cancer	13.5		12.5	1720.5	.02
Menstruation	Pregnancy	13.5		12.75	38,180	.007
Procedure	General disease	12.75		12.25	7886.5	.02
Procedure	Cancer	12.75		12.5	2422.5	.93
Procedure	Pregnancy	12.75		12.75	54,094	.56
General disease	Cancer	12.25		12.5	2747.5	.06
General disease	Pregnancy	12.25		12.75	62,984	<.01^a^
Cancer	Pregnancy	12.5		12.75	26,927	.64

^a^*P*<.005.

^b^RQ4: research question 4.

## Discussion

### Principal Findings

This study applied 4 TRMs to score the readability of OB/GYN online PEMs and compared the scores of the TRMs, which is the first to conduct such comparative analysis and provide empirical evidence to show the limitations of the TRMs. The findings showed that the 4 TRMs lacked agreement with one another, indicating the need for developing modern readability measures for PEMs and general OHI. Using the average score of TRMs as a proxy to the readability level, our study found that most OB/GYN PEMs required high readability levels despite the recommendation for online PEMs to be at the sixth-eighth–grade reading level. In addition, the study found that the online PEMs from the government source were slightly easier to read than other sources. However, they still require 11th grade level. It is important to provide health information in a consistent language so that OHI can be disseminated to all, regardless of source. Lastly, the comparison of the topic categories showed that the menstruation and pregnancy PEMs are harder to read than the general disease. This opens new research questions and research investigations.

### Implications

The finding where the 4 TRMs did not agree with one another connects well with recent research concerning TRMs. For example, 1 study evaluating the readability of diabetes-related PEMs found that certain readability measures consistently indicated higher levels of readability than others [22]. This indicates that the existing measures do not reflect completely the readability level of health texts [29]. Moving away from syntax and surface language features, recent research has identified other factors, such as sentence complexity, use of passive voice, grammar frequency, and the patient’s familiarity with vocabulary, as additional components that need to be considered in measuring health text readability [30-32]. Passive voice, in particular, was shown to distinguish very complex texts from very simple texts, but was not able to distinguish intermediate complexities, making it an important language feature to study further [33]. Additionally, the evaluation of readability measures is critical, especially using human annotations since the readability measures may not reflect the actual readability and understandability of a piece text. Previous studies have found such discrepancy between the perceived document difficulty and actual difficulty [19,34]. Our study highlights the need to improve the readability of OB/GYN PEMs and ensure the validity and reliability of readability measures.

When looking at readability assessment studies done in medical specialties other than OB/GYN, required readability levels are consistently very high [35-37], showing a need for remediation across specialties like ophthalmology, cardiology, geriatric care, and beyond. For example, in 1 study specifically using TRM to analyze ophthalmology related PEMS, average readability was found to be at the 10th-grade reading level [35]. Even with inconsistent TRM levels, human annotations have rated PEMs in several medical fields to be too difficult to understand [38]. Such studies have been repeated in various specialties over several years, highlighting the issue of readability in health education as a pertinent issue for some time.

Over the years, some studies have moved away from the use of TRM, most likely due to their poor ability to accurately assess health information. Recently developed readability assessment methods have adopted machine learning, artificial intelligence, and NLP techniques, which have been shown to better predict readability when compared to human annotations [29,39,40]. However, these methods have not been widely used or promoted in the research community. With further advancement of artificial intelligence and NLP, such as pretrained large language models, it may become easier to assess readability, and even revise and generate easier-to-read health information automatically.

### Limitations

This study has at least 6 limitations. First, the PEM search may not be comprehensive; it may miss other online PEMs in the field of OB/GYN. However, we used modern search engines to collect PEMs that were most likely to be accessed by laypersons. Therefore, the PEMs included in this study should be frequently seen by laypersons. Since the study only included the results on the first page of each search engine, relevant websites may be listed on the second page or after. Second, the PEMs were scored by only 4 TRMs. There are other TRMs (such as Fry readability graph [41] and FORCAST [42]) that could potentially be more useful and better adept at identifying the true readability of online PEMs. Moreover, there have been other readability measures developed recently for health information [19,29,31]. However, since many of the new readability assessment methods have not been widely used by the research community, they were excluded in this study. Third, in this study, the texts for all PEMs were taken without any content analysis, which can limit our understanding of the actual readability level. Fourth, although statistical significance was found in the results, there is not sufficient evidence to prove that small differences in grade level above grade 12 are meaningful since most readability measures are calibrated for school grades. Next, although the PEMs were cleaned, the title and some sections of the PEMs were removed, which may affect the readability score distribution [43]. Lastly, the search results might have been slightly altered due to the presence of cache and other confounders in Google search [44].

### Future Work

Future work includes surveying the literature and summarizing the current advances in readability formula development, given the fact that although research has been conducted to create new readability formulas, very few are promoted and widely used to replace TRMs in the research community. Implementing these health information specific readability measures may provide some more insight into what must be done to improve the readability of online PEMs. Additionally, there will be further content analysis conducted on PEMs to create writing guidelines to support clinicians and patients alike, especially in the context of OB/GYN. Patient stakeholders or standardized patients can also inform the creation of new PEM content for validation of health care–specific readability tools. Lastly, a simple English thesaurus for OB/GYN PEMs can be developed to help those writing PEMs to simplify their materials.

### Conclusions

This study examined the readability of the PEMs in OB/GYN. While the OB/GYN PEMs were collected using search engines, which may introduce biases, the study found that most of the PEMs were hard to read, requiring high school or college level of education to read the content. More research is needed to evaluate the PEMs in OB/GYN in a more comprehensive manner and create writing guidelines to improve the readability, understandability, and actionability of these PEMs.

## Appendix

Multimedia Appendix 1 The PEM website source information and categorization.

## Abbreviations

AMA: American Medical Association
DCL: Dale-Chall
FKGL: Flesch-Kincaid grade level
GFI: Gunning fog index
NIH: National Institutes of Health
NLP: natural language processing
OB/GYN: obstetrics and gynecology
OHI: online health information
PEM: patient education material
SMOG: Simple Measure of Gobbledygook
RQ1: research question 1
RQ2: research question 2
RQ3: research question 3
RQ4: research question 4
TRM: traditional readability measures
