# Supplementary material for: Assessing the Readability of Online Patient Education Materials in Obstetrics and Gynecology Using Traditional Measures: Comparative Analysis and Limitations
Source: J Med Internet Res. 2023 Aug 30;25:e46346. doi: 10.2196/46346 (PMC10500363; doi:10.2196/46346)
Supplement: Multimedia Appendix 1 [file jmir_v25i1e46346_app1.docx]

**Multimedia Appendix 1.** The PEM website source information and categorization.

| Source name | Source URL | Source categorization | Count** |
| --- | --- | --- | --- |
| Medline Plus | <https://medlineplus.gov/womenshealth.html> | Governmental | 135 |
| Patient Education - Ascension Center for Women's Health | <https://acwh.net/patient-education/> | Commercial | 34 |
| Patient Literature Library \| Epocrates | <http://www.epocrates.com/patientresources/category/1216> | Commercial | 16 |
| Southdale ObGyn Clinic- Infertility Patient Education | <https://www.southdaleobgyn.com/infertility/new-infertility-patient-education-materials/> | Commercial | 1 |
| OB/GYN Patient Education | <https://www.massgeneral.org/obgyn/patient-resources/> | Educational | 5 |
| Patient Education Materials \| USF Health | <https://health.usf.edu/care/obgyn/patient-ed-materials> | Educational | 4 |
| ClassicCity | <http://ih-cco.eznetpublish.ihealthspot.com/Home/PatientEducationLibrary/tabid/20468/Default.aspx> | Commercial | 66 |
| Women's Health \| ACOG | <https://www.acog.org/Womens-Health> | Commercial | 132 |
| Patient Education Genius* | <https://dashboard.coherentrx.com/> | Various Categories | 1,183 |

*This source includes American Pregnancy Association (Nonprofit), U.S. Food and Drug Administration (Governmental), March of Dimes (Nonprofit), MotherToBaby (Nonprofit), Endometriosis.org (Educational), Mayo Clinic (Nonprofit), and Top OB/GYN Drugs (Commercial).

**The total count is 1,576.
